# Butterfly Larval Host Plant use in a Tropical Urban Context: Life History Associations, Herbivory, and Landscape Factors

**DOI:** 10.1673/031.011.6501

**Published:** 2011-05-21

**Authors:** Ashish D. Tiple, Arun M. Khurad, Roger L. H. Dennis

**Affiliations:** ^1^ Entomology Division, Department of Zoology, RTM Nagpur University, Nagpur-440 033, India; ^2^ Forest Entomology Division, Tropical Forest Research Institute, Jabalpur- 482021, (M. P.) India; ^3^ Centre for Ecology and Hydrology, Wallingford, Maclean Building, Benson Lane, Crowmarsh Gifford, Wallingford, Oxon OX10 8BB, UK, and Institute for Environment, Sustainability and Regeneration, Staffordshire University, Mellor Building, College Road, Stoke-on-Trent ST4 2DE, UK. School of Life Sciences, Oxford Brookes University, Headington, Oxford OX3 0BP, UK

**Keywords:** India, Lepidoptera, habitat, host specificity, life form, biotope, perennation, oviposition, vegetation structure, landforms

## Abstract

This study examines butterfly larval host plants, herbivory and related life history attributes within Nagpur City, India. The larval host plants of 120 butterfly species are identified and their host specificity, life form, biotope, abundance and perennation recorded; of the 126 larval host plants, most are trees (49), with fewer herbs (43), shrubs (22), climbers (7) and stem parasites (2). They include 89 wild, 23 cultivated, 11 wild/cultivated and 3 exotic plant species; 78 are perennials, 43 annuals and 5 biannuals. Plants belonging to Poaceae and Fabaceae are most widely used by butterfly larvae. In addition to distinctions in host plant family affiliation, a number of significant differences between butterfly families have been identified in host use patterns: for life forms, biotopes, landforms, perennation, host specificity, egg batch size and ant associations. These differences arising from the development of a butterfly resource database have important implications for conserving butterfly species within the city area. Differences in overall butterfly population sizes within the city relate mainly to the number of host plants used, but other influences, including egg batch size and host specificity are identified. Much of the variation in population size is unaccounted for and points to the need to investigate larval host plant life history and strategies as population size is not simply dependent on host plant abundance.

## Introduction

Insects have unrivalled supremacy among living organisms constituting, as they do, the largest faunal component inhabiting the earth, occupying almost all ecological niches, from the frozen Arctic and Antarctica, to dry deserts, hot springs and high mountains. Among insects, butterflies have proved to be invaluable flagship species for conservation (Thomas 2005). Confronted with worldwide pressures on natural biomes, from an exponentially growing human population, they have already been shown to be highly sensitive indicators of climate change (Parmesan et al. 1999; Sparks et al. 2005 2007), biotope fragmentation (Warren et al. 2001) and urbanization (Hardy and Dennis 1999; Jana et al. 2006; Kadlec et al. 2008). Most research findings emanate from temperate environments, yet, a wealth of butterfly data is potentially available for monitoring changes to tropical biomes. For instance, India hosts about 1,501 butterfly species, 350 in peninsular India and 333 in the western Ghats alone (Gaonkar 1996).

Butterflies are phytophagous. The ability of herbivorous insects to feed on plants has been demonstrated to be intricately linked to plant taxonomic diversity (Mitter et al. 1988) and involves competition between plants and insects (Dawkins and Krebs 1979). The dominant strategy among herbivorous insect species involves specialization on a set of closely related plants (Ehrlich and Raven 1965; Eastop 1973; Ehrlich and Murphy 1988; Ward and Spalding 1993). Butterfly-plant speciation, through shifts in host-plant ranking and specialization, is thought to account for a substantial part of the diversification of plant-feeding insects (Thompson et al. 1990; Carriere and Roitberg 1995; Keese 1996; Janz 1998; Janz and Nylin 1998). All herbivorous insects show some degree of host selectivity (Bernays and Chapman 1994). Under natural conditions, insects are confronted with many external stimuli, their own internal physiological conditions and responses, and a series of environmental constraints (Visser 1986; Bernays and Chapman 1994; Badenes et al. 2004). This makes it very difficult to discern the relative importance to the insect of chemical, visual, and mechanical stimuli from host and non-host plants (Schoonhoven et al. 1998; Hooks and Johnson 2001). However, it is generally assumed that the process of host selection in specialist insects is governed primarily by volatile chemical signals, later by visual stimuli, and finally by non-volatile chemical signals (Hern et al. 1996; Hooks and Johnson 2001). Butterflies demonstrate a hierarchy in host preferences, discriminating among plant species, among genotypes, among individuals with different phenological and physiological conditions, and even among plant parts (Wiklund 1984), although not all discriminate at the finer scales (Wiklund 1975; Thompson and Pellmyr 1991; Bernays and Chapman 1994). Furthermore, there may be significant behavioural differences within a family, among species of the same genus, or even among different populations of the same species (Jones 1977; Singer and Parmesan 1993). Many butterflies prefer groups of very closely related plants where the larvae obtain the entire set of nutrients required for growth and development, as well as chemicals for display (colours) and defence as adults (Boppré 1984).

Thus, the relationship between any given butterfly species and its host plant is very specific. Among all the resources required by butterflies that comprise a habitat (Dennis et al. 2003 2006; Dennis 2010), the larval host plants are the key resource, being fundamental for reproduction. Knowledge of butterfly host plants is a prerequisite for any butterfly conservation programme. Therefore, it is necessary to know the exact needs of the immature stages to make conservation successful (New et al. 1995). But, knowledge concerning larval host plants is still poor in the case of many butterfly species, especially in the tropics (Kunte 2000). As such, the present study focuses on larval host plant use in the butterflies of biotopes within the confines of Nagpur City, India, building on the work of previous scientists.

In central India butterfly species diversity has been investigated by D'Abreeu (1931) who documented 177 species within the previous Central Provinces (now Madhya Pradesh and Vidarbha). In addition to this D'Abreeu (1931) provided a list of 92 butterfly species from Nagpur city. More recently, Pandharipande (1990) has recorded 61 species of butterflies from Nagpur city. Several objectives or lines of inquiry have been made. First, a database has been constructed including larval host plants for butterflies resident in a range of biotopes within the city. Second, an investigation has been made of interrelationships between different aspects of herbivory in relation to major taxa (butterfly families and subfamilies) of butterflies. Finally, relationships have been sought between general abundance of butterflies and herbivory (host plant and host use factors). It is expected that the population size of butterfly species will reflect basic differences in their major consumer resources and that these differences will extend to contrasts at higher taxonomic levels. Such contrasts are basic to conservation strategies for the butterfly fauna; this study aims to collect necessary information for the formulation of butterfly conservation management plans.

## Materials and Methods

### Study sites

The study was conducted in and around Nagpur, central India (20° 99′ N, 79° 99′ E) by one of us (ADT; data are available from the first author), between 1 June 2006 and 31 May 2008, as part of a wider study on butterfly diversity of Nagpur City. Nagpur is the second city of Maharashtra state; it is located on the Deccan plateau in central India. The original biome in this area was dry deciduous forest dominated by *Tectona grandis* (teak), *Diospyros melanoxylon* (tendu leaves) and various species of *Terminalia* trees. Nagpur has a tropical dry equable climate marked by three distinct seasons: a very hot and dry summer (March to May), a wet season during which most of the precipitation occurs with the south-western monsoon (June–September), and a mild winter (November to February; October being the post-monsoon transitory period). The total mean annual precipitation is ∼1,100mm, the annual average temperature 27° C, and the annual average humidity 51% (Tiple et al. 2009).

Data on oviposition, larval feeding and butterfly numbers were collected from six sites in Nagpur (Table 1); the latter were obtained from extensive Pollard transect records (Pollard and Yates 1993; Tiple et al. 2009b) over the sites, each divided into three transect sections (each 500 m long). The sites differ in biotopes (vegetation structure) and in resources for butterflies (i.e., occurrence of larval host plants, flowering nectar plant species and physical structures used for oviposition and breeding). The relative abundances of butterfly species taken over all sites in Nagpur (27,700 individuals, minimum 15, maximum 1575 individuals per species), distinguished where possible by sex, were obtained from the transect records taken within confined bounds (5 metre square area) walked at a steady pace (Tiple et al. 2009b). Although transect counts do not provide absolute estimates of butterfly populations and, owing to their different biotope associations and conspicuousness to recorders, are not directly comparable (Dennis et al. 2006b), the large range in numbers obtained for different species are regarded here as adequately reflecting relative differences in population sizes of butterfly species. Oviposition and breeding records, as well as nectar use and plant distributions, were obtained during independent surveys of the same sites (Tiple et al. 2009b, Tiple et al. 2010).

### Rearing of caterpillars and pupae

During the survey one of us (ADT) followed female butterflies and collected the eggs along with the plant parts on which eggs were laid. The foliage was also searched, along with other plant parts, for eggs and larvae. The larvae observed during the survey were collected and brought to the laboratory along with their host plant leaves for rearing. The cages containing larvae were cleaned daily before old foliage was replaced by fresh leaves. Following larval growth and pupation, the pupae were left in the cages undisturbed until adult eclosion, when they were identified. Although some larvae and broods were lost to mortality, larvae were often sufficiently distinct to identify to species level (Table 2).

### Identification of plants

The larval host plants were identified and noted together with their butterfly larvae and adults. Those plants that were difficult to identify in the field were preserved by making dry herbarium sheet specimens including all details of the plants for further identification. These herbarium specimens were identified in consultation with Prof. K. H. Makde and Dr. N. M. Dongerwar, Department of Botany, RTM Nagpur University, Nagpur, and other knowledgeable taxonomists.

### Larval host plant variables

Butterfly species and larval host plants were scored for a number of variables considered to influence herbivory. Butterfly species were distinguished for specificity (phagy) into monophagous (feeding on one plant family) and polyphagous (feeding on more than one plant family) (*sensu* Veenakumari et al. 1997). Butterfly species were also scored for ant associations (ant protection versus no recorded association) and egg laying batch size (1 single or few eggs each batch, 2 small batch of 5 to 20; 3 large batch of 20 to 100, 4 very large batch of eggs each time > 100). From a previous survey (Tiple et al. 2009b) butterfly species were also scored for joint use of plants as a nectar source and larval food (0 no, 1 yes). Host plants were scored for plant growth form or habit (H herb, S shrub, T tree, C climber, P stem parasite), biotope (W wild, C cultivated, E exotic), abundance (R rare, F frequent, A abundant) and perennation (A annual, B Biannual, P perennial). Data on plant ecotone and edge distributions had previously been obtained during a survey of mate location behavior. These variables include common occurrence of the host plant: along herb or shrub track edges, along shrub or woodland edges, at rock face or wall, along stream or river bank and on hill tops (each binary coded, 0 no, 1 yes).

### Analysis

Multiple correspondence analysis (MCA) has been used to examine relationships among host plant, life history variables and butterfly taxa. For examination of associations (correlations) and/or multiple correspondence analysis, a number of the variables were recoded to binary or ranked scales (plant growth form: 1 herb, 2 shrub, 3 tree, climber and epiphyte; biotope: 1 wild, 2 cultivated and exotic; host plant abundance: 1 rare or frequent, 2 abundant; egg batch number: 1 single/small < 5 eggs, 2 large and very large > 5 eggs). Significance of direct associations within MCA plots is reported as nonparametric Kendall's tau (τ). Kendall's tau is equivalent to the phi coefficient √(χ^2^/N), the latter applied to categorical or binary data (Siegel 1956). The analysis of butterfly population size in relation to host plant and host use factors applied a general linear regression model with transect counts log transformed and the residuals tested for normality.

**Figure 1.**
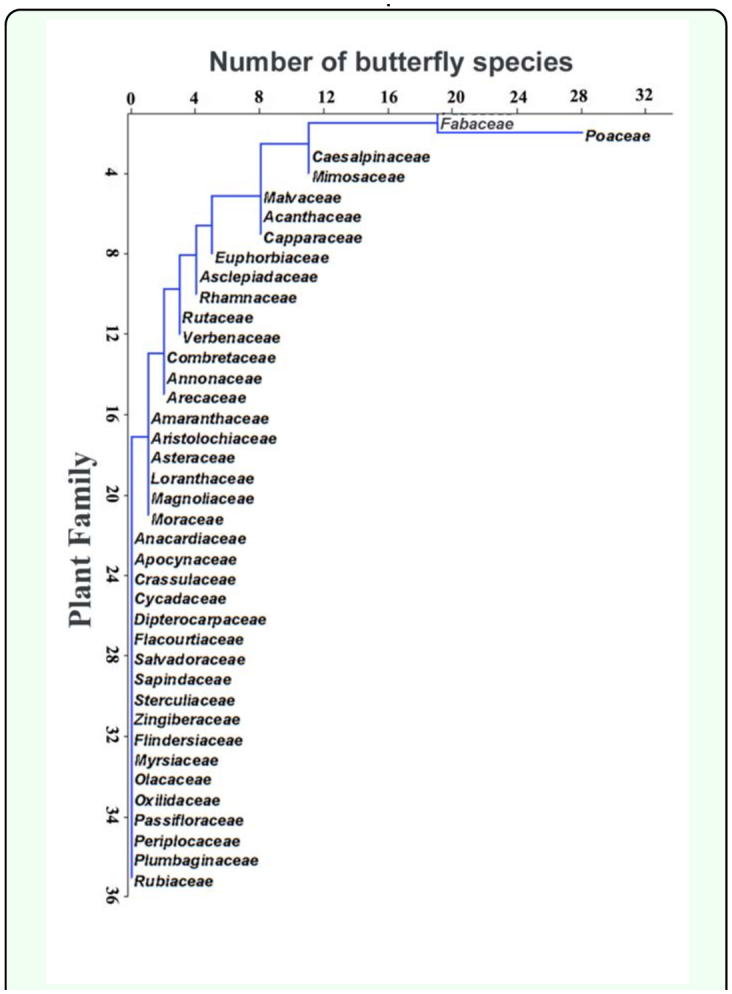
Plant families preferred by the butterflies for larval feeding and development. High quality figures are available online.

## Results

### Larval host plant database

Of some 145 butterfly species recorded in and around Nagpur city, the larval host plants of 120 species of butterflies belonging to five families were identified. Altogether, 124 larval host plants were listed. A host plant list for butterflies is provided in Table 2 and attributes for the host plants in Table 3. A new larval host plant *Chloroxylon swietenia* for *Papilio demoleus* was found for this area.

Among the 120 butterfly species, eight were Papilionidae, 17 Pieridae, 46 Nymphalidae, 30 Lycaenidae and 19 Hesperiidae. Most of the butterfly species were monophagous (*sensu* Veenakumari et al. 1997) (24 butterfly species feed on only one plant species, 24 butterfly species feed on one plant genus and 40 butterfly species feed on more than one plant genus (but confined to one family) n = 88), the remaining 32 butterfly species were polyphagous (Table 2). The 126 host plants include 89 wild (native) plant species, 23 that are cultivated, 11 that are native but cultivated and 3 species that are exotic (plant species which are not native to India). The plants varied substantially in life form (habit). Most are trees (n = 49), followed by herbs (43), shrubs (22), climbers (7) and stem parasites (2). Most of the plants are perennials (n = 78), a smaller number annuals (n = 43) and few biannuals. The majority of the plants were abundant (n = 88) at the Nagpur sites, but a substantial number (n = 27) were not abundant and nine plant species were observed to be rare.

### Taxonomic associations in host use

The five butterfly families were found to use host plants from 39 plant families at Nagpur. No significant differences were found in the number of plant families used by butterfly families (χ^2^_4_ = 0.82, p = 0.93). The plants belonging to Poaceae (29) and Fabaceae (20) families were found to be the most widely used by butterfly larvae (Figure 1). Over a dozen butterfly species' larvae fed on each of these two families in this region. Twelve butterfly species fed on plants in families Mimosaceae and Caesalpinaceae, nine species on each of the Acanthaceae, Capparaceae and Malvaceae and six on Euphorbiaceae. The larvae of Papilionidae had a preference for Rutaceae, Pieridae for Capparaceae and Caesalpiniaceae, Nymphalidae prefered Acanthaceae and Malvaceae and Lycaenidae, Fabaceae and Mimosaceae. Both Nymphalidae (specifically the subfamily Satyrinae) and Hesperiidae had a preference for Poaceae (Table 2). Other butterfly families overlap in the use of host plants from the same plant family but to a lesser extent (Table 4). Among plant species, the grasses were an exclusive larval host for 13 butterfly species, followed by *Barleria prionitis* for nine species, *Capparis zeylanica* for six species, *Sida cordifolia* for five species, and *Bambusa* spp., *Calotropis gigantean, Hibiscus* spp., *Pongamia pinnata* and *Tephrosia purpurea* each for 4 butterfly species.

### Associations among host plant and butterfly biology variables

**Figure 2.**
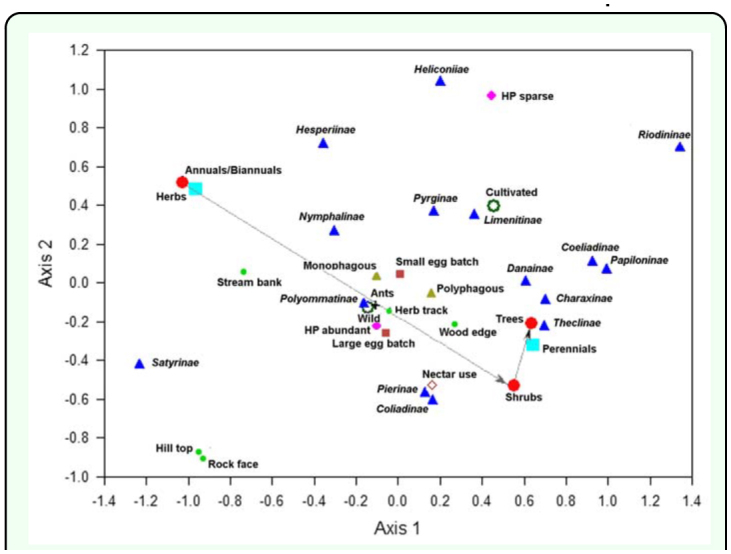
Multiple correspondence analysis of larval host plant attributes for Nagpur butterflies. Axis 1 28% inertia, axis 2 16% inertia. Active variables: host plant life form (large open circles), host plant biotope (large closed circles), host plant abundance (open diamond), host plant perennation (large closed squares), landform affiliations including tracks through herbs/shrubs [Herb track], Shrubwood edge [Wood edge], rock face, stream banks and hill top (small open circles); supplementary variables: host plant used as nectar source [nectar use] (closed diamond), phagy (open triangle), egg batch size (small open square), ant association [Ants] (cross), taxa (subfamilies) (large closed triangles): Papilionidae (Papilioninae), Pieridae (Coliadinae, Pierinae), Riodinidae (Riondininae), Lycaenidae (Polyommatinae, Theclinae), Nymphalidae (Nymphalinae, Satyrinae, Limenitinae, Danainae, Charaxinae, Heliconiinae), Hesperiidae (Hesperiinae, Pyrginae, Coeliadinae). Analysis weighted by numbers of host plants. The arrow indicates increasing trend in vegetation succession for sites. High quality figures are available online.

Associations among the host plant variables are presented in Table 5 and Figure 2. The multiple correspondence analysis (Figure 2) is based on integer codes and weighted over different host plants, whereas conservative estimates of significance for Kendall's τ have been calculated over the means of ranks for species. Ignoring Bonferroni corrections some 29 of 87 correlations were significant; 19 of 78 with use of a Bonferroni correction. As expected, larval host plant life form (habit) is closely associated with plant perennation as a consequence of higher growth forms (trees) being perennials. Higher growth forms are also significantly associated with shrub-wood edges. Lower growth forms (herbs) are more closely associated with stream banks, hill tops and rock faces. The simple biotope division of wild versus cultivated host plants produces different associations; wild plants are associated with hill tops and paths through herbs. Larval host plant abundance increased on hill tops, rock faces and stream banks. Perennials were associated with shrub and woodland, including shrub-wood edges, whereas annuals increased on rock faces, stream banks and hill tops. Plants used as nectar sources tend to be higher life forms (shrubs and trees), but this relationship was significant only when species were weighted for numbers of host plants (Kendall τ = 0.13, P = 0.0035, n = 233).

**Figure 3.**
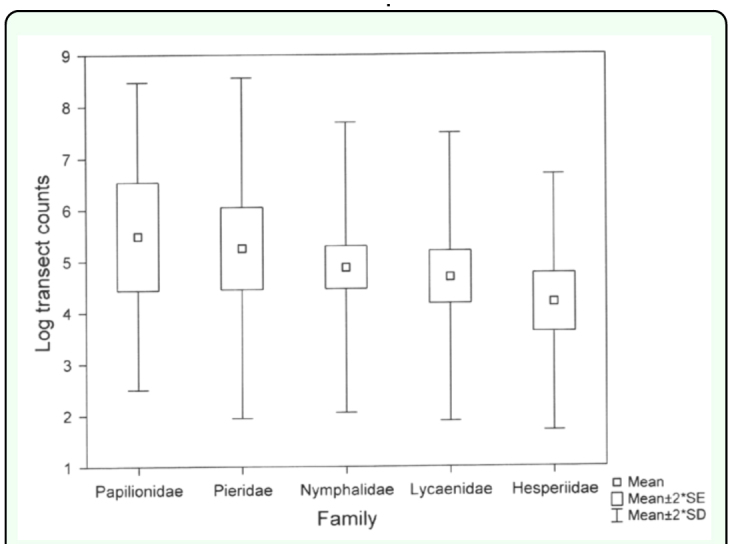
Comparison of transect counts for butterfly families (means for species) at all sites. High quality figures are available online.

Egg batch size and ant association increased significantly with polyphagy, the latter case was significant after Bonferroni correction. Ant association also increased with perennial taller plants, but decreased for plants at the shrub-wood edge.

The relative use of annual plants was found to increase with the number of host plants on which species were found (relative frequency of annuals and biannuals to total plants, Kendall τ = 0.12, P = 0.048, n = 120). The relationship was stronger when species were placed into two groups, those using one host plant and more than one host plant (Kendall τ = 0.17, P = 0.007, n = 120). Some 14 of 48 species feeding on only a single host plant used annuals (29.2%) whereas 39.7% of plants used by butterfly species feeding on more than 1 plant were annuals.

### Taxonomic contrasts in host use and herbivory

Significant contrasts among butterfly families occur for host use of different host plant life forms (χ^2^_(4)_ = 36.60, P < 0.0001), biotopes (χ^2^_(4)_ = 40.83, P < 0.0001), host plant perennation (χ^2^_(4)_ = 21.60, P < 0.001) but not for host plant abundance (χ^2^_(4)_ = 3.99, P = 0.40). Nymphalidae and Hesperiidae used more herbs than expected, whereas Papilionidae and Lycaenidae used more trees and Pieridae more shrubs than expected. An excess of Nymphalidae and Hesperiidae host plants occured wild compared to an excess of Hesperiidae and Papilionidae that were cultivated/exotic. Corresponding with these contrasts, an excess of Nymphalidae and Hesperiidae used annuals/biannuals, whereas Papilionidae, Lycaenidae and, to a lesser extent, Pieridae, used more perennials than expected. Families also differed for host specificity (phagy) (χ^2^_(4)_ 9.75, P = 0.045) with Hesperiidae having a significant tendency towards monophagy and Lycaenidae towards polyphagy. Finer taxonomic divisions (subfamily level) occur as illustrated in Figure 2.

Landscape contrasts among host plants for butterfly families occured for stream banks (χ^2^_(4)_ = 29.19, P < 0.0001) and hill tops (χ^2^_(4)_ = 11.63, P = 0.02) but not shrub-wood edges (χ^2^_(4)_ 8.83, P = 0.065). An excess of Nymphalidae and Hesperiidae host plants were found on stream banks, and a deficit of host plants belonging to Papilionidae and Pieridae. Hill tops had an excess of Pieridae and Nymphalidae host plants and a deficit of Papilionidae and, to a lesser extent, Hesperiidae host plants. The number of absences were too small for a comparison of host plant occurrence along tracks through herbs and shrubs for all families, but an excess of Hesperiidae occured along tracks compared to those of Nymphalidae and Lycaenidae, the latter two not differing in frequency (χ^2^_(1)_ = 10.70, P = 0.001).

Numbers were too small for tests of host plant nectar use by adults and egg batch size, although eight of 13 species producing large egg batches were Pieridae. All 26 species with ant associations were Lycaenids.

### Butterfly abundance and herbivory

A comparison of log transect counts taken over all sites indicates no overall distinction in transect counts among butterfly families (F(4, 115) = 1.83, P = 0.13) although a Fisher LSD post hoc test produced a significant difference between Hesperiidae and both Papilionidae (P = 0.033) and Pieridae (P = 0.0028) (Figure 3). Transect counts correlated significantly with five host plant and herbivory related variables (Kendall τ, number of host plants 0.37 P < 0.0001, phagy 0.23 P = 0.0002, egg batch number 0.21 P = 0.0008, tracks through herbs 0.19 P = 0.003, stream banks -0. 18 P = 0.004), with numbers increasing except at stream banks. In general the regression model of log transect counts against all host plants, life history and herbivory variables (stepwise forwards entry) two variables accounted for significant amounts of variation, the number of host plants and tracks through herbs and shrubs (F_(117)_ = 20.04, R^2^ = 0.26, P < 0.0001) (Table 6).

## Discussion

The basic objective of the Nagpur study was the construction of a database on resources for butterflies to further their conservation (cf., Dennis et al. 2008). A database allows progress in two important areas. First, it supplies firm information on resources and resource use by butterflies; secondly, it provides the means for identifying taxonomic contrasts for and interactions among life history and ecological variables to ensure that resources are allocated in an efficient, holistic manner to conserve and build butterfly communities in suitable sites. The current paper on larval host plants and herbivory is the fourth in the study, the former three exploring adult feeding and population dynamics (Tiple et al. 2009a, b) and mate location resources and resource use (Tiple et al. 2010). Larval host plants are the prime consumer resource. Without them, butterfly species are incapable of building populations. The current study identifies 124 host plants for 120 butterfly species and documents aspects of host plant life history. The outcome has been the disclosure of substantial, significant differences in host use and herbivory among higher taxonomic units (families), important links between host plants and herbivory variables and insights into contrasting population abundances among species.

### Resources and resource use

The study has focused on collecting fundamental information of butterfly resources within Nagpur City, India. Data on the other vital consumer resource, nectar flowers (Tudor et al. 2004) have already been reported (Tiple et al. 2006, 2009b). Basic information has been collected on host plant life forms, basic biotopes, perennation, abundance, and host plant ecotone/edge distributions. Of 126 larval host plants, most were trees (49) followed by herbs (43), with fewer shrubs (21), climbers (7) and stem parasites (2). Regarding basic biotopes, 89 of the larval host plants were plants growing wild, 23 were cultivated, 11 originally native now cultivated and 3 exotic plant species. There was a clear ranking in the importance of different plants for butterfly species. The plants belonging to Poaceae (29) and Fabaceae (20) families were found to be the two most widely used by butterfly larvae. With regard to perennation, 76 plants were perennials, 43 annuals and 5 biannuals. The host plants of most species (n = 88, 70%), from a simple audit, were found to be abundant, but a substantial number (n =27) are less so (frequent) and nine plant species were recorded as rare in and around Nagpur city. Out of 120 butterfly species, 88 butterfly species were currently found to be monophagous and remaining 32 butterfly species were polyphagous, for the strict criteria of plant family level distinction.

The prominence of particular plant families as butterfly host plants, in part, reflects the overlap in their use by species of different families. Thus, Poaceae are important for both Hesperiidae and Nymphalidae (Satyrinae), and the Fabaceae by species of both Lycaenidae and Nymphalidae. Veenakumari et al., (1997) also reported that Poaceae and Fabaceae were found to be the most widely exploited plant families by butterfly larval stages in the Andaman and Nicobar islands. Ackery (1991) also found the Poaceae and Fabaceae to be the predominant larval host families. The Poaceae were dominant; the grasses were an exclusive larval host for 13 butterfly species followed by *Barleria prionitis* (Acanthaceae) which caters for nine butterfly species.

The dominant use of perennial plants at Nagpur is not surprising. Soloman Raju et al. (2003) made similar observations on Andhra University campus, Vishakhapatnam, where butterfly species showed preference to perennial plants. This observation is strongly favored by annual conditions of growth for higher, woody plants; at the beginning of summer (December – January) perennial trees shed their leaves and sprouting initiates at the same time. The young leaves survive the hot summer and at the onset of monsoon (June– July) trees produce luxuriant growth in terms of leaves until the following dry season and leaf loss. The number of annual plants used at Nagpur is high compared to that found in temperate butterfly communities (Kemp et al. 2008). This is not surprising. Climatic conditions of Nagpur city are almost ideal for butterfly development and the continual production of annual plants on disturbed ground given sufficient moisture; this provides better opportunities for butterfly species to lay their eggs during all seasons and increased chances for the survival of larval stages. The inclusion of 43 annual plants in the host plant resource bank for species highlights the role of smaller, native plants growing wild in comparison to perennial trees for supporting the life cycle of butterflies.

The frequency of monophagous species (73%) in the current study is high and it is expected that supplementary host plants may be found in future studies within the study site. However, Soloman Raju et al. (2003) and Veenakumari et al. (1997) produced similar findings and reported that most of the butterfly species were monophagous and very small number were polyphagous. Monophagy has potentially serious implications for conservation; in the absence of supplementary host plants, monophagous butterfly species depend on abundance and ubiquity of the sole host plant. In fact, as expected from resource theory (Dennis 2010), butterfly species that were polyphagous had more host plants (Kendall τ = 0.51, P < 0.0001) than those that are monophagous. They may well have greater overall abundances of host plant cover regardless of differences in mean abundances among the actual plants. In the Nagpur study, mean abundance of host plants was greater for polyphagous species than monophagous species but not significantly so (mean 1.86 versus 1.76, medians both 2.0; Kendall τ = 0.07, P = 0.24, n =120).

### Taxonomic contrasts in host use and life history

Although overlap amongst higher taxa emerges in larval host plant use, thus in host plant attributes and landscape associations (e.g., Satyrinae and Hesperiinae in use of grasses and herb rich areas), more notable are contrasts between higher taxa for host use and herbivory. Such are the bias of plants in Nagpur; Nymphalidae and Hesperiidae to herb rich areas and Papilionidae, Lycaenidae and Pieridae to shrub-rich and tree-rich biotopes. This distinction at Nagpur corresponds to an excess of Nymphalidae (Satyrinae and Nymphalinae) and Hesperiidae (Hesperiinae) host plants occurring wild and being annuals compared to an excess of Hesperiidae (Coeliadinae) and Papilionidae host plants that are cultivated/exotic and perennial (Figure 2). Species belonging to different families contrast for life history attributes. Obvious ones include differences for host specificity (e.g., tendency for Hesperiidae to be monophagous and Lycaenidae to be polyphagous), egg batch size (e.g., more Pieridae have large egg batches) and ant association, which is restricted to the Lycaenidae. These differences are compounded by associations between vegetation, landscape and life history variables. For instance, perennation was correlated with life form (plant habit) and both life forms and basic biotope distinctions (wild versus cultivated plants) have distinct links to vegetation structures and landforms: wild plants with hill tops and tracks through herbs, cultivated plants with shrubs, trees and wood edges. Among life history variables, egg batch size and ant association were found to correlate with phagy (polyphagy), though the former was not supported by a Bonferroni correction of significance. Such contrasts translate into zonation of higher taxa within distinct landscape elements, with an excess of Nymphalidae and Hesperiidae host plants along stream banks, Hesperiidae along tracks through herbs and shrubs, and Pieridae and Nymphalidae host plants on hill tops. These differences produce heterogeneity in butterfly communities for different landforms and vegetation structures and are of paramount importance in planning conservation measures for butterflies within the city confines.

### Factors influencing butterfly population sizes

A prominent area for research is investigation of factors that underlie general differences in population sizes among species. An examination of the different factors affecting population sizes over the different sites in Nagpur City will be the subject of a later paper. Here, we consider the factors briefly that influence overall differences in population size among species. There is an assumption that transect counts accurately portray differences in population size. As it is, transect counts are affected by conspicuousness of species to recorders and this can potentially affect counts along transects (Dennis et al. 2006b).

Even so, two life history variables clearly have an impact on transect counts: egg batch size and host specificity (phagy), which are mutually correlated. Species which lay larger egg batches and feed on plants from different families, have larger transect counts and very probably have larger populations. The latter variable, phagy, also correlates closely with number of host plants used (Kendall τ = 0.51, P < 0.0001, n = 120). The number of host plants has been found previously to be a key variable in population size in a temperate context, accounting for 22% of the variation in transect counts (Dennis et al. 2004 2005). This is not surprising; supplementary host plants provide a sound theoretical basis for increased population size (Dennis 2010). What is interesting is that none of the other life history, herbivory or vegetation landscape features account for much additional variation once number of host plants has been entered into regression equations (tracks through herbs, an additional 4%); it is at first surprising that there is no significant effect of differences among butterfly families or that host plant abundance does not have a significant influence. Although, differences in conspicuousness of species to recorders may account for some additional variation, it will not account for the unexplained 73% of variation. Previously, Dennis et al. (2004) have pointed to the importance of other host plant life history factors that it has not been possible to assess in the current study; it is worth investigating if host plant strategies drive butterfly status in tropical regions and ascertaining the prominence of the C-S-R strategy model for plants (Grime 1974 1979; Price 2002; Hunter 2003) in this urban area. It also has to be recalled that in a resource-based definition of habitat that butterflies use other resources and will spread over other areas to gain adult food, to mate, roost and engage in other resource exploitation (Dennis 2010)

### Implications for conservation

Not all butterflies at Nagpur have the same conservation status. Among the 120 butterfly species 20 species come under the protection category of the Indian Wild Life (protection) Act 1972. Among them *Neptis jumbah, Actolepis puspa, Amblypodia anita, Pachliopta hector, Lethe europa, Neptis columella, Castalius rosimon, Hypolimnas misippus* are addressed under schedule I of the act. The species recorded which come under schedule II are *Eurema andersonii, Appias albina, Euthalia aconthea, Cepora nerissa, Pareronia Valeria, Melanitis zitenius, Euchrysops cnejus, Lampides boeticus, Jamides celeno*, and those recorded which come under schedule IV are *Appias libythea*, *Tarucus ananda, Euploea core* (Gupta and Mondai 2005; Kunte 2000). Their persistence at Nagpur is undoubtedly dependent on the maintenance of reported larval food plants.

Of the 20 butterfly species under the highest protection category, 10 species are monophagous (*Pachliopta hector, Eurema andersonii, Appias libythea, Appias albino, Pareronia Valeria, Melanitis zitenius, Lethe europa, Neptis columella,, Tarucus ananda, Amblypodia anita*) and the remainder polyphagous (*Cepora nerissa, Neptis jumbah, Euthalia aconthea, Hypolimnas misippus, Euploea core, Castalius rosimon, Actolepis puspa, Euchrysops cnejus, Lampides boeticus, Jamides celeno*). The plants for the 10 monophagous butterfly species are abundant with the notable exception of *Aristolochia indica*. One of the primary factors likely responsible for the healthy status of butterflies at Nagpur is that 88 larval host plants were found to be abundant; diversity for larval host plants, especially wild plants in natural contexts, is undoubtedly a key variable in maintaining butterfly diversity within the city area. It is inevitable that there has been a decline in both larval host plants and nectar plants in Nagpur City during its recent development. During last decade the dimensions of the city have doubled threatening the loss of natural biotopes of butterflies. Urban development is expected to have a deleterious impact on butterfly populations, if only because the construction of buildings, tarmac and concrete replaces or reduces the area of natural and semi-natural biotopes. The quality of residual habitats may also be adversely affected by various forms of pollution (Tiple et al. 2007; Tiple and Khurad 2009). The main message is a simple one: as wild plants are crucial for maintaining butterfly diversity, it is vital to conserve them and their biotopes; identifying relationships between butterfly taxa and host plant variables (Figure 2), as done for butterfly taxa and nectar flower variables (Tiple et al. 2009b), provides a useful foundation for generating ‘green spaces’ for butterflies within the city environment. Expanding suburbia, more intensive development of agriculture land and plantation of exotic species, are all significant threats.

Many butterfly species come under direct protection. Yet, not all rare species are formally protected and may well require protection following a reappraisal of their status. We consider this a matter of urgency. Knowledge of their larval host plants and other resources, the development of a resource databank for species (Dennis et al. 2008), will take us at least part of the way in formulating effective conservation management programs for them.

**Table 1.**
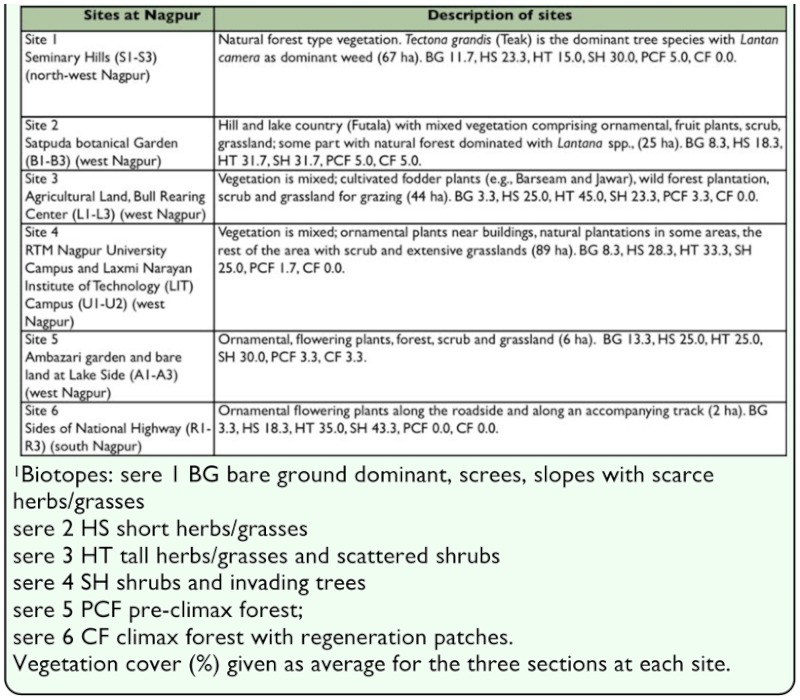
Sites for data collection in Nagpur City, India.

**Table 2.**
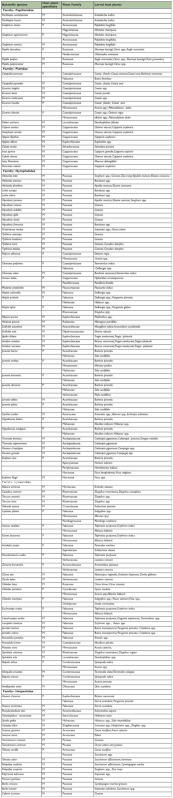
Butterfly species larval host plants and their specificity (M- monophagous and P- polyphagous).

**Table 3.**
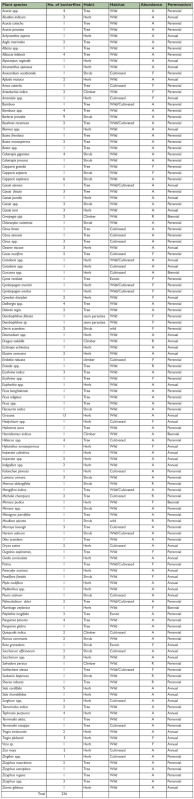
Larval host plants, their habit, habitat, abundance and perennation (A- abundant, F- frequent, R- rare).

**Table 4.**
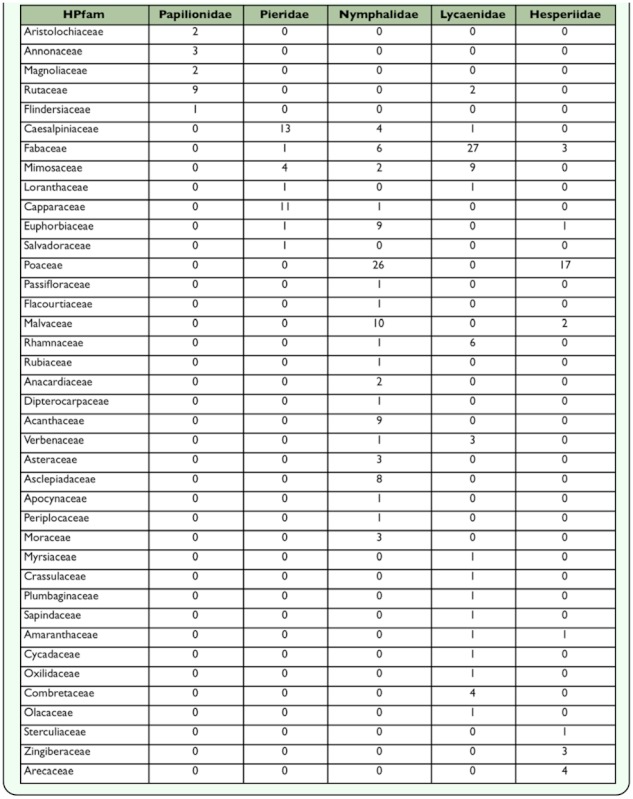
Exploitation of plant families as larval host plants by species of butterfly families at Nagpur, India.

**Table 5.**
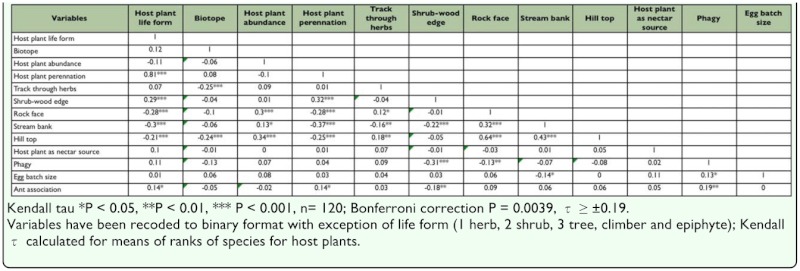
Correlations (Kendall τ between butterfly larval host plant variables, host plant specificity (phagy) and egg batch size.

**Table 6.**
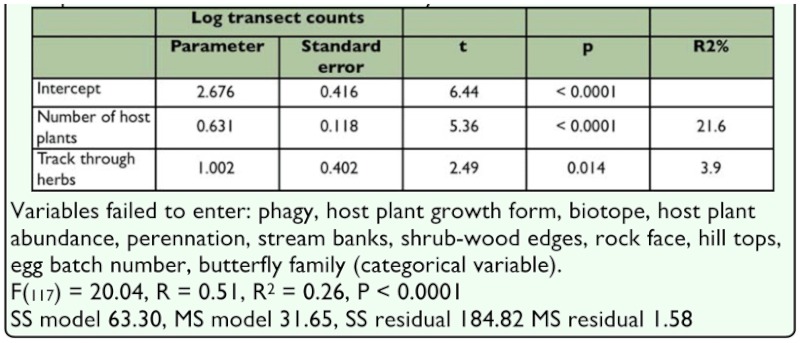
General linear model of transect counts for butterfly species against host plant variables controlled for butterfly families.
